# Severe *Isospora (Cystoisospora) belli* Diarrhea Preceding the Diagnosis of Human T-Cell-Leukemia-Virus-1-Associated T-Cell Lymphoma

**DOI:** 10.1155/2012/640104

**Published:** 2012-08-16

**Authors:** N. Ud Din, P. Torka, R. E. Hutchison, S. W. Riddell, J. Wright, A. Gajra

**Affiliations:** ^1^Department of Medicine, SUNY Upstate Medical University, 750 East Adams Street, Syracuse, NY 13210, USA; ^2^Department of Pathology, SUNY Upstate Medical University, 750 Adams Street, Syracuse, NY 13210, USA

## Abstract

*Isospora (Cystoisospora) belli* diarrhea can sometimes be fulminant in immunocompromised patients. It is endemic in tropical and subtropical areas, and sporadic episodes have been reported in nonendemic areas in nursing homes, day-care centers, and psychiatric institutions. We describe isosporiasis in an HIV-negative Sudanese-American female who presented with a debilitating diarrheal illness and profound weight loss. *Isospora belli* was detected in her stool by modified acid-fast staining. Serologic testing was negative for HIV but positive for HTLV-1 infection. Treatment with TMP-SMZ led to improvement in her diarrhea which recurred after stopping antibiotics. Subsequently, she developed generalized lymphadenopathy which was diagnosed as ATLL on immunohistochemical staining. Chemotherapy was initiated, but her condition continued to worsen due to persistent diarrhea and resulting profound electrolyte abnormalities. The patient opted for comfort measures and died a few weeks later at a nursing facility. This case emphasizes that the detection of *I. belli* should trigger testing for HIV, HTLV-1, and other causes of immunocompromise. We suggest that treatment with TMP-SMZ should be initiated and continued for a prolonged period of time in immunocompromised patients with *I. belli* diarrhea.

## 1. Introduction

*Isospora belli* is a coccidian, unicellular protozoan parasite that resides in the gastrointestinal tract. It usually causes nonbloody diarrhea in tropical and subtropical climates [1, 2]. In developed countries, it is found in recent immigrants, travelers returning from endemic regions, and patients with AIDS [3]. The disease course is mild and usually transient in immunocompetent hosts. In immunocompromised individuals, the disease can vary in severity from a chronic intermittent illness to severe life-threatening diarrheal illness. We describe severe isosporiasis in a non-HIV patient with human T-cell-leukemia-virus-1- (HTLV-1-) associated T-cell lymphoma living in a nontropical climate. 

## 2. Case

A 44-year-old Sudanese-American female was transferred to our hospital from another facility with the chief complaint of chronic diarrhea. She had emigrated from Sudan to the United States ten years previously and had never revisited her home country. She was relatively healthy until nine months prior to admission when she experienced the insidious onset of cramping epigastric pain and diarrhea. The diarrhea was in large volume, with 10–20 bowel movements daily. Her stools were watery, with very little formed stool and no blood or mucus. Her condition led to profound weakness and debility, and she was essentially bed-bound. She reported intermittent nausea, vomiting, severe loss of appetite, and a 100-pound weight loss in the preceding nine months. She denied any fever, sick contacts, history of foreign travel, hiking, camping, exposure to animals, or drinking well water. Past medical history was significant for a positive tuberculin test ten years ago. She was breast feeding at that time and therefore did not receive isoniazid. She denied smoking but admitted drinking 6 beers per day until just prior to her presentation.

Over the preceding nine months, the patient had been admitted to a community hospital several times and extensively investigated for the cause of her diarrhea. Stool ova and parasite examination, as well as *Giardia* and *Cryptosporidium* antigen tests, was negative. A workup for malabsorption and colonoscopy was normal; upper GI endoscopy showed mild gastritis and blunting of small intestinal villi. The patient was started on a celiac diet with no improvement in her symptoms. HIV and hepatitis serologies were negative. Her thyroid function tests were normal. A CT scan of the abdomen showed fatty liver.

**Figure 1:**
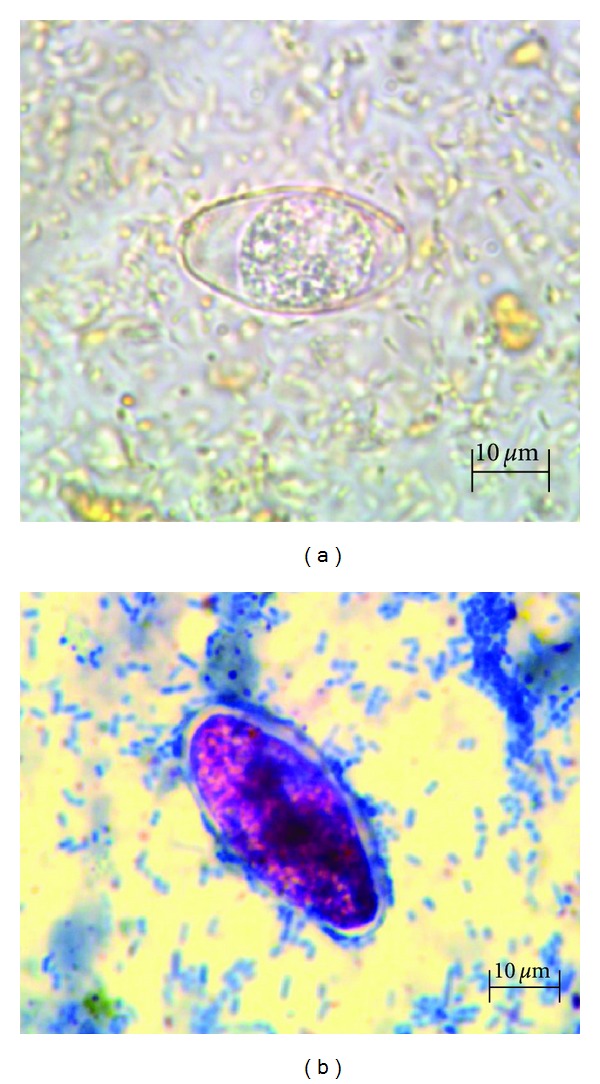
(a) *Isospora belli*, saline mount. (b) *Isospora belli*. Modified acid-fast stain.

Upon transfer to our hospital, the patient was found to be severely dehydrated with multiple electrolyte abnormalities: sodium 133 mmol/L, potassium 4.0 mmol/L, chloride 105 mmol/L, bicarbonate 14 mmol/L, BUN 2.85 mmol/L, creatinine 30.50 mmol/L, magnesium 0.6 mmol/L, and phosphorus 1.13 mmol/L. She was anemic with a hemoglobin of 94 g/L and hematocrit of 31%. Her leukocyte count was 10.3 × 10^9^/L with 64% neutrophils and an increased absolute lymphocyte count of 2.16 × 10^9^/L; the rest of the differential was normal. Erythrocyte sedimentation rate was more than 120 mm/hr. Albumin was 25 g/L, AST 64 U/L, ALT 104 U/L, alkaline phosphatase 187 U/L, total bilirubin 5.13 *μ*mol/L, and direct bilirubin 3.42 *μ*mol/L. Diarrhea was again evaluated, and tests for ova and parasites, including microsporidia, stool cultures, polymerase chain reaction (PCR) for *Clostridium difficile*, fat and reducing substances, osmolar gap, fecal leukocytes, and occult blood, were all negative. Anti-tissue transglutaminase IgA and anti-gliadin IgA antibodies were negative. Vasoactive intestinal peptide and urine 5-HIAA levels were normal. Consultation with gastroenterology led to an additional stool culture for *Salmonella, Shigella, Campylobacter, Aeromonas, Plesiomonas,* and* E. coli* O157, repeat ova and parasite exam, and tests for *Cryptosporidium *antigen*, Cyclospora,* and *Isospora*. All testing was negative with the exception of a modified acid-fast stain which was positive for *Isospora (Cystoisospora) belli* (Figure 1).

An infectious disease consult suggested investigating for potential causes of immunocompromise as the degree of diarrhea was too severe for a simple *I. belli* infection. A repeat HIV 1, 2, and HIV group *O* test was negative by serology. There was no evidence of immunoglobulin deficiency; serum IgA was normal, while IgG and IgM were mildly elevated. The patient was treated with trimethoprim 160 mg (TMP)-sulfamethoxazole 800 mg (SMZ) four times a day for two weeks with resolution of diarrhea. 

**Figure 2:**
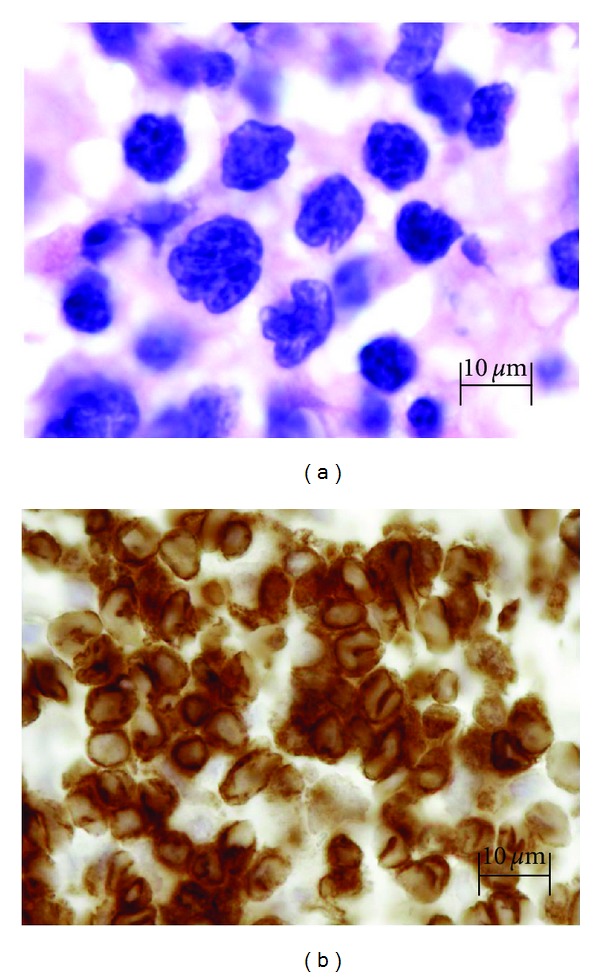
(a) Right femur shavings showing aggregates of small-to-medium-sized lymphoid cells with irregular to convoluted nuclei, with admixed large cells and eosinophils. Hematoxylin and eosin. (b) Immunohistochemical stains showing T-cell phenotype. CD3.

Unfortunately, the abdominal pain and loss of appetite persisted and a subsequent HTLV-1 and 2 antibody screen was reported positive. HTLV-1 infection was confirmed by western blot. At followup one month later, the patient was found to have new bilateral inguinal lymphadenopathy. A CT scan of the chest showed axillary, mediastinal, and right hilar lymphadenopathy. Likewise, a CT scan of the abdomen showed new extensive retroperitoneal lymphadenopathy which was enclosing and displacing the vasculature. There was a lytic lesion in the right femoral neck with significant fragility of the cortex as well as sclerotic abnormality of the L3 transverse process and L3 vertebral body. Skeletal survey and bone scan showed multiple lytic lesions. A right inguinal lymph node biopsy showed partial effacement by an interfollicular T-cell infiltrate with residual B-cell nodules mimicking follicular lymphoma. Immunohistochemistry showed the abnormal cells to express CD2, CD3, and CD4 with absence of CD5 and CD7. Flow cytometry confirmed the immunophenotype and PCR for T-cell receptor gamma gene showed a monoclonal rearrangement. B-cell gene rearrangement assay was polyclonal. Biopsy of the right hip and femur curettings showed involvement by peripheral T-cell lymphoma with expression of CD3, CD25, and partial CD30 (Figure 2).

 A diagnosis of ATLL was rendered as per WHO guidelines [4]. Her diarrhea returned in the interim, and repeat stool examination was positive for *I. belli*, prompting a repeat course of TMP-SMZ.

The patient was transferred to the oncology service and started on the cyclophosphamide, hydroxydaunorubicin hydrochloride (doxorubicin hydrochloride), vincristine, and prednisone (CHOP) regimen. An orthopedics consult for right hip pain led to prophylactic stabilization of the right femur for an impending pathologic fracture. Femoral shavings revealed a focal atypical lymphoid infiltrate consistent with lymphoma. The patient then underwent two cycles of treatment with CHOP for stage 4 T-cell lymphoma. She did not tolerate chemotherapy well. Abdominal pain and diarrhea continued despite TMP-SMZ; nausea and vomiting did not improve significantly. She returned to the hospital several times over the next three months with dehydration, severe electrolyte imbalances, and complications secondary to chemotherapy and concomitant loss of weight and strength. After discussion with the patient and her family, a mutual decision was taken to send her to a nursing home with hospice care. The patient died a few weeks after discharge.

## 3. Discussion

*Isospora (Cystoisospora) belli* is a coccidian parasite that invades the small intestinal epithelium and completes its life cycle in the cytoplasm of the enterocyte. Oocysts (diagnostic stage) are excreted in the feces and develop outside the host into mature cysts, each with two sporocysts which in turn contain four sporozoites each (infective stage). Humans are the only known hosts*. I. belli* infection usually causes loose stools and watery diarrhea which can rarely be fulminant [5]. The diarrhea is often associated with colicky abdominal pain, fever, nausea, vomiting, malabsorption, weight loss, and peripheral eosinophilia. The infection is typically self-limited in immunocompetent hosts but rarely can be chronic. Sporadic outbreaks have been reported in mental institutions and day-care centers as well [6]. However, it is an opportunistic pathogen which causes protracted wasting diarrhea in patients with HIV/AIDS. The prevalence of *I*.* belli* infection was estimated to be 15% of AIDS patients in Haiti but is <0.2% in the US AIDS population likely secondary to prompt treatment of recurrences with TMP-SMZ (as well as routine use of TMP-SMZ for *Pneumocystis carinii *prophylaxis [1]) [5, 7]. It has also been reported in other immunosuppressive conditions such as adult T-cell leukemia, acute lymphoblastic leukemia, Hodgkin's lymphoma, and thymoma [8–11]. The organism is endemic to the tropics, especially Africa and the Middle East where the incidence ranges from 0.2% to 20% in patients with AIDS. It has also been reported from Argentina, Haiti, India, Japan, Trinidad, and Taiwan. In a large retrospective study from India [12], *I. belli* was found in 0.1% of 1029 cancer patients with diarrhea. All but one of these cases were associated with HTLV-1 seropositivity. 

This case is unusual because the patient had been living in the United States for the last ten years, and we found only one other similar case in the medical literature. Resiere et al. described a patient from Mali with non-Hodgkin's lymphoma who had been living in France for eight years before presenting with *I. belli* infection. However, in contrast to our case, their patient was HTLV-1 and 2 negative and was already on chemotherapy before the infection became manifested [13]. In addition, the amount of weight loss experienced by our patient was the highest among the cases of *I. belli* diarrhea identified in our search. This weight loss may have resulted from the combined effects of chronic diarrhea and the underlying malignancy.

*I. belli* is usually transmitted by the ingestion of contaminated food or water. The diagnosis is usually made by detecting oocysts by wet-mount microscopy or modified acid-fast staining; these structures may also be visualized through the property of autofluorescence [7, 14–16]. As with other intestinal parasites, multiple specimens may be required to demonstrate the organism, and for light infections, biopsy of the mucosa of the small intestine may be required. Nucleic acid amplification assays have been described but are not widely available [17, 18]. 

Isosporiasis responds well to treatment. A 7–10-day course of TMP-SMZ provides rapid clinical and parasitological cure [19]. Other treatment regimens include pyrimethamine-sulfadoxine and nitazoxanide, although there is limited data to support this. Fluoroquinolones can be used; however they have been found to be less efficacious when compared to TMP-SMZ [20]. Diarrhea frequently recurs in AIDS patients; hence, secondary prophylaxis is recommended, using either TMP-SMZ three times weekly or sulfadoxine-pyrimethamine weekly [5]. Repeated stool microscopy at monthly intervals may be done to ensure eradication of infection. There are no such guidelines for patients with malignancies; however based on our patient's fulminant course, we would suggest prophylaxis for these patients until their immunosuppression resolves. 

In conclusion, detection of *I. belli* in stool samples in patients with severe diarrhea should trigger an aggressive search for causes of immunocompromise such as HIV or hematologic malignancies. A thorough history should be obtained regarding travel to endemic areas, hiking, camping, drinking well water, or residence in institutional settings. Tests for HTLV-1 and 2 antibody must be part of the routine workup, especially in individuals living in developed countries as it can cause immunocompromise similar to HIV. Immunocompromised individuals should be treated with a prolonged course of TMP-SMZ and routine prophylaxis should be considered as well. In patients with HTLV-1-positive T-cell lymphoma, examination for *I. belli *and TMP-SMZ prophylaxis during the entire course of chemotherapy should be considered in patients with recurrent or protracted episodes of diarrhea.
